# Whether mindfulness-guided therapy can be a new direction for the rehabilitation of patients with Parkinson’s disease: a network meta-analysis of non-pharmacological alternative motor-/sensory-based interventions

**DOI:** 10.3389/fpsyg.2023.1162574

**Published:** 2023-09-14

**Authors:** Shenglan He, Wanyi Fang, Jiaoyang Wu, Hang Lv, Jueyu Zhang, Tunyi Wang, Yingjie Huang, Guangyao Li, Min Li

**Affiliations:** ^1^Medical College of Acupuncture Moxibustion and Rehabilitation, Guangzhou University of Chinese Medicine, Guangzhou, China; ^2^The Affiliated Traditional Chinese Medicine Hospital of Guangzhou Medical University, Guangzhou, China; ^3^Guangdong Second Traditional Chinese Medicine Hospital (Fifth Clinical Medical College of Guangzhou University of Chinese Medicine), Guangzhou, China

**Keywords:** mindfulness-guided therapy, Parkinson’s disease, qigong, network meta-analysis, non-pharmacological interventions

## Abstract

**Background:**

The treatment for Parkinson’s disease (PD) consumes a lot of manpower and financial resources. Non-pharmacological alternative motor-/sensory-based interventions are optimized for the rehabilitation of PD patients. Mindfulness-based therapy shows ideal efficacy, but the diversity of the therapy brings difficulties to the selection of clinicians and patients.

**Methods:**

Network meta-analysis in the Bayesian framework was used to evaluate the efficacy of non-pharmacological alternative motor-/sensory-based interventions in improving motor and non-motor symptoms in PD patients.

**Results:**

A total of 58 studies (2,227 patients) were included. Compared with the non-intervention group, qigong was associated with improved outcomes in the Timed Up and Go (TUG) test (mean difference (MD) −5.54, 95% confidence interval (CI) −8.28 to −2.77), and UPDRS-I (MD −15.50, 95% CI −19.93 to −7.63). Differences between non-pharmacological alternative motor-/sensory-based interventions were not significant for PDQ-39, UPDRS-I, or UPDRS-II; however, qigong was superior to dance (MD −3.91, 95% CI −6.90 to −0.95), Tai Chi (MD −3.54, 95% CI −6.53 to −0.69), acupuncture (MD −6.75, 95% CI −10.86 to −2.70), music (MD -3.91, 95% CI −7.49 to −0.48), and exercise (MD −3.91, 95% CI −6.49 to −1.33) in the TUG test.

**Conclusion:**

This network meta-analysis supports mindfulness-based therapy (e.g., qigong, yoga, and Tai Chi) as a preferred non-pharmacological alternative motor-/sensory-based intervention for PD rehabilitation.

**Systematic review registration:**

https://inplasy.com/inplasy-2022-10-0109/, INPLASY2022100109.

## 1. Introduction

Parkinson’s disease (PD) is the second most prevalent progressive neurodegenerative disease among middle-aged and elderly people worldwide (Prasad and Hung, 2021) and is estimated to affect more than 12 million people by 2050, making it the fastest-growing neurological disease in the world. Patients with PD can mainly suffer motor and non-motor symptoms. Among them, four typical motor symptoms, including bradykinesia, tremor at rest, rigidity, and postural instability, are considered to be the main components of this disease (Readinger et al., 2021). Non-motor symptoms include fatigue, sleep disorders, depression, anxiety, olfactory and gustatory dysfunction, cognitive impairment, and autonomic regulation disorders (Wu et al., 2021). The treatment and rehabilitation of large numbers of PD patients can be labor-intensive and expensive, and therefore, economical and effective therapies are urgently required (Osborne et al., 2022).

Current pharmacological treatments for PD are mainly divided into dopaminergic and non-dopamine drugs (Armstrong and Okun, 2020). The U.S. Food and Drug Administration approves levodopa as the most common drug for the treatment of PD because it readily crosses the blood–brain barrier to increase dopamine concentrations (Oliveira de Carvalho et al., 2018; Prasad and Hung, 2021). However, levodopa can easily cause motor complications such as chorea or stereotypy and wear-off dystonia when peak doses are reached (Espay et al., 2018). In addition, these most commonly used drugs are not effective in preventing or delaying disease progression (Fox et al., 2018). Furthermore, the underlying pathology or side effects associated with drugs can bring non-motor symptoms to PD patients and should be considered as well (Mooventhan and Nivethitha, 2017).

Recently, an increasing number of clinicians have focused on complementary and alternative therapies for PD. Non-pharmacological alternative motor−/sensory-based interventions such as qigong, yoga, Tai Chi, dance, acupuncture, music, and exercise have been commonly carried out in PD, and it is of concern that mindfulness-based therapies such as qigong, yoga, and Tai Chi seem to exert notable efficacy (Zhang et al., 2022). Research has shown that qigong practice increases resting-state functional connectivity (rs-FC) between the hippocampus and the medial prefrontal cortex and improves overall cognitive function in PD patients (Wang et al., 2022). Mindfulness yoga has been shown to be effective in treating Parkinson’s disease-related psychological and motor symptoms in recent randomized controlled trial (RCT) studies, and its effect may be related to psychoneurotic immune markers (such as cortisol and cytokines; Kwok et al., 2019). Although people differ in their attitude toward the practice of mindfulness-guided therapy (Tang et al., 2015, 2016), some researchers believe that training in mindfulness meditation can reduce amygdala reactivity and enhance amygdala-ventromedial prefrontal cortex (VMPFC) connectivity, thus improving the ability to regulate the emotions of the brain (Kral et al., 2018). However, other studies have shown that no meditation program has an impact on positive mood or attention (Goyal et al., 2014). It can be seen that the dispersion of evidence greatly reduces the reliability of these therapies. This study performed a network meta-analysis (NMA) to compare the efficacy of non-pharmacological measures in treating PD, and to the best of our knowledge, the meta-analysis may be the first indirect comparative study of non-pharmacological alternative motor−/sensory-based interventions of PD. The purpose of this study was to investigate the advantages and disadvantages of different interventions on the Unified Parkinson’s Disease Rating Scale (UPDRS), the Timed Up and Go (TUG) test, and the Parkinson’s Disease Quality of Life Questionnaire (PDQ-39), and we hope that the conclusions obtained from this meta-analysis can break the dilemma of PD treatment to some extent and provide a substantial reference to the rehabilitation of patients with PD.

## 2. Methods

### 2.1. Protocol and registration

According to Preferred Reporting Items for Systematic Reviews and Meta-Analyses (PRISMA) 2020 guideline, the first reticular meta-analysis study of non-pharmacological alternative motor−/sensory-based interventions for PD was conducted and we registered the protocol in the International Platform of Registered Systematic Review and Meta-analysis Protocols (INPLASY; registration number: 2022100109).

### 2.2. Literature review

We searched PubMed, Cochrane Library, Embase, Web of Science, and EBSCO CINAHL Ultimate up to October 2022 under the subject headings and random word searches with terms for acupuncture (including various acupuncture techniques), Tai Chi, qigong, yoga, dance, music, exercise, meditation, and mindfulness. Relevant core journals and available books from libraries were also searched manually. The language was limited to English.

### 2.3. Inclusion criteria

The study selection was based on the following inclusion criteria: (1) participants diagnosed with PD following the criteria of the International Parkinson and Movement Disorder Society or stating a clear diagnosis of the disease and following their medication regimen; (2) interventions with non-pharmacological alternative motor-/sensory-based interventions such as acupuncture (including various acupuncture techniques), Tai Chi, qigong, yoga, music, exercise, mindfulness, and meditation (motor-/sensory-based mindfulness or meditation therapies are included), in combination with basic PD drugs; (3) control defined as pharmacological, placebo, or non-intervention, or non-motor interventions; (4) outcomes that included at least one of the UPDRS, the TUG test, and PDQ-39; and (5) controlled clinical trials. Search strategies are shown in Supplementary Figure 1.

### 2.4. Exclusion criteria

Excluded studies had the following characteristics: (1) PD patients mixed with other neurodegenerative diseases; (2) pure mindfulness and meditation therapies that were not exercise-based; (3) reports, reviews, clinical protocols, conference abstracts, and animal experiments; (4) unable to download; (5) unknown sample size, irrelevant results, or data dropouts; and (6) repeated publications.

### 2.5. Study selection

**Figure 1:**
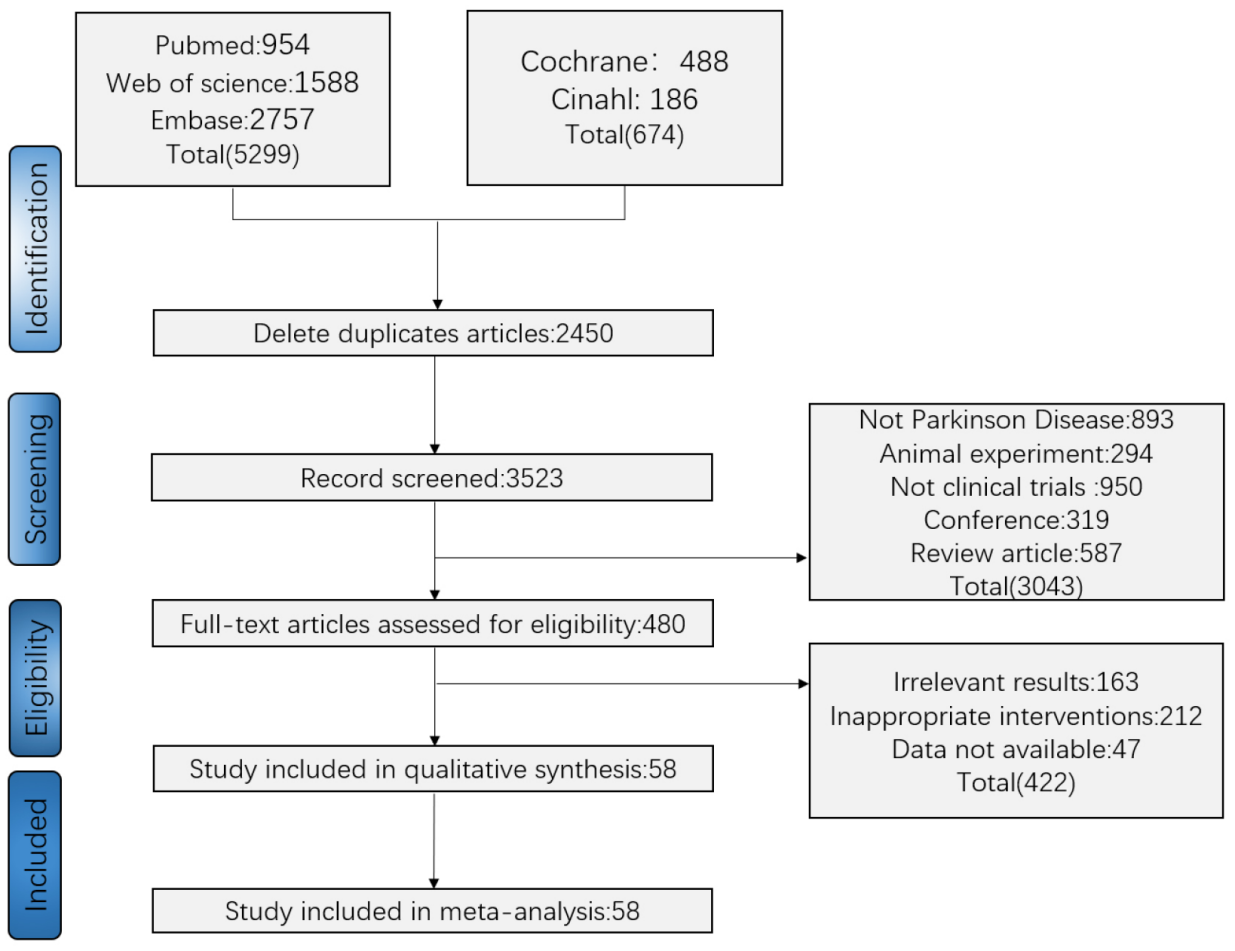
Screening process.

All retrieved studies were imported into Endnote X9 software with duplicates removed. According to the order of the inclusion/exclusion criteria, two researchers (SLH and WYF) independently screened relevant studies by title and abstract. Any disputes were resolved by consulting a third assessor (JYW) through discussion or arbitration (Figure 1).

### 2.6. Data extraction and outcome measures

Data were extracted independently by two researchers into Microsoft Excel 2019, including first author, country, intervention (sample size and characteristics), course of treatment, and outcome measures. All discrepancies were resolved by consensus after a re-review of the study by all authors. The primary efficacy outcomes were the TUG test, UPDRS-III, and PDQ-39; and the secondary efficacy outcomes included the UPDRS overall score, UPDRS-I, and UPDRS-II.

### 2.7. Risk-of-bias assessment

The Cochrane risk bias assessment tool was used to evaluate the included articles, including random sequence generation, allocation concealment, blinding of participants and personnel, blinding of outcome assessment, incomplete outcome data, and selective reporting.

### 2.8. Data synthesis and evaluation of the statistical assumption

Continuous variables were presented as mean differences (MD) with 95% confidence intervals (CI). We used Stata 14 and Aggregate Data Drug Information System (ADDIS) 1.16.8 to perform NMA aggregation employing a Markov chain Monte Carlo method under a Bayesian-based framework according to the PRISMA-NMA instructions. The estimated effect was used to predict the ranking, and the probability was expressed according to the value of the histogram. In the node-splitting model, ADDIS used the consistency model when *p* > 0.05, and the non-consistency model when *p* < 0.05. The consistency model can assess the effect size and calculate the ranking of the interventions. The comparison was statistically significant (*p* < 0.05) when the 95% CI did not include 0.

## 3. Results

### 3.1. Characteristics of the included studies

**Figure 2:**
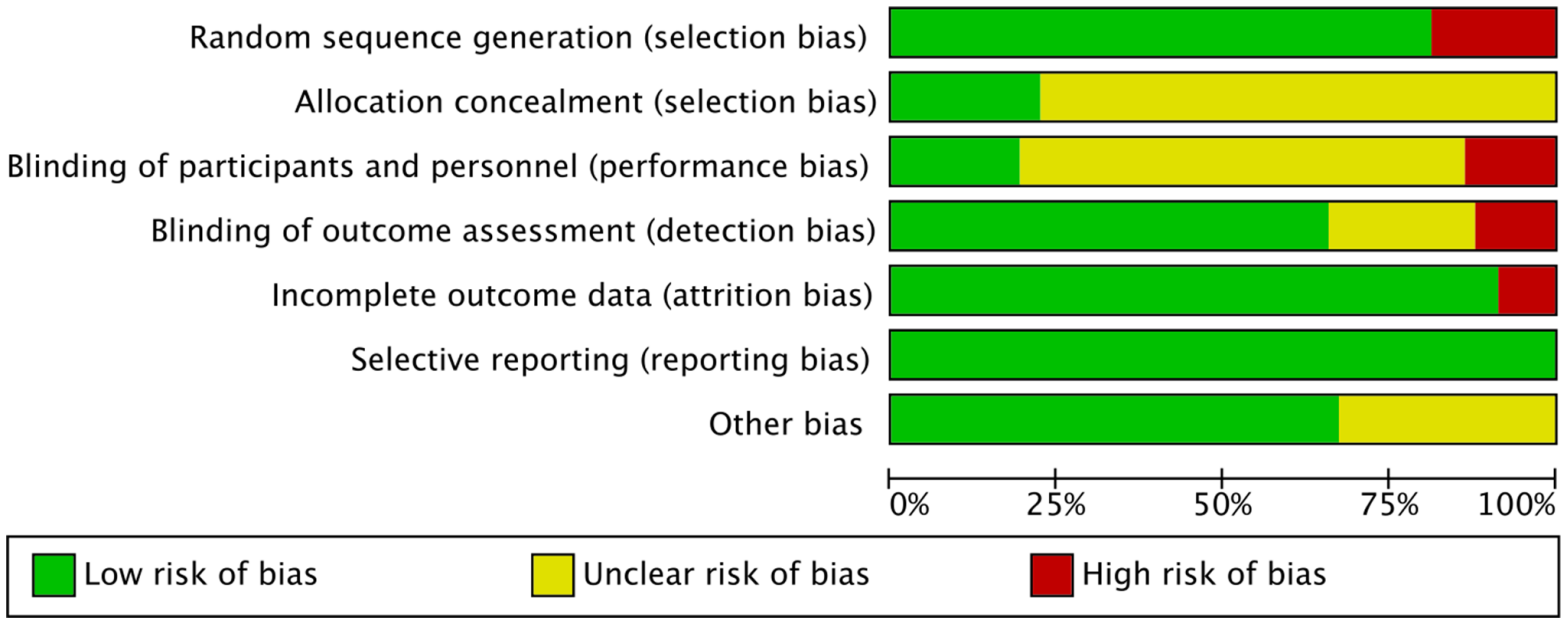
Risk-of-bias graph.

**Figure 3:**
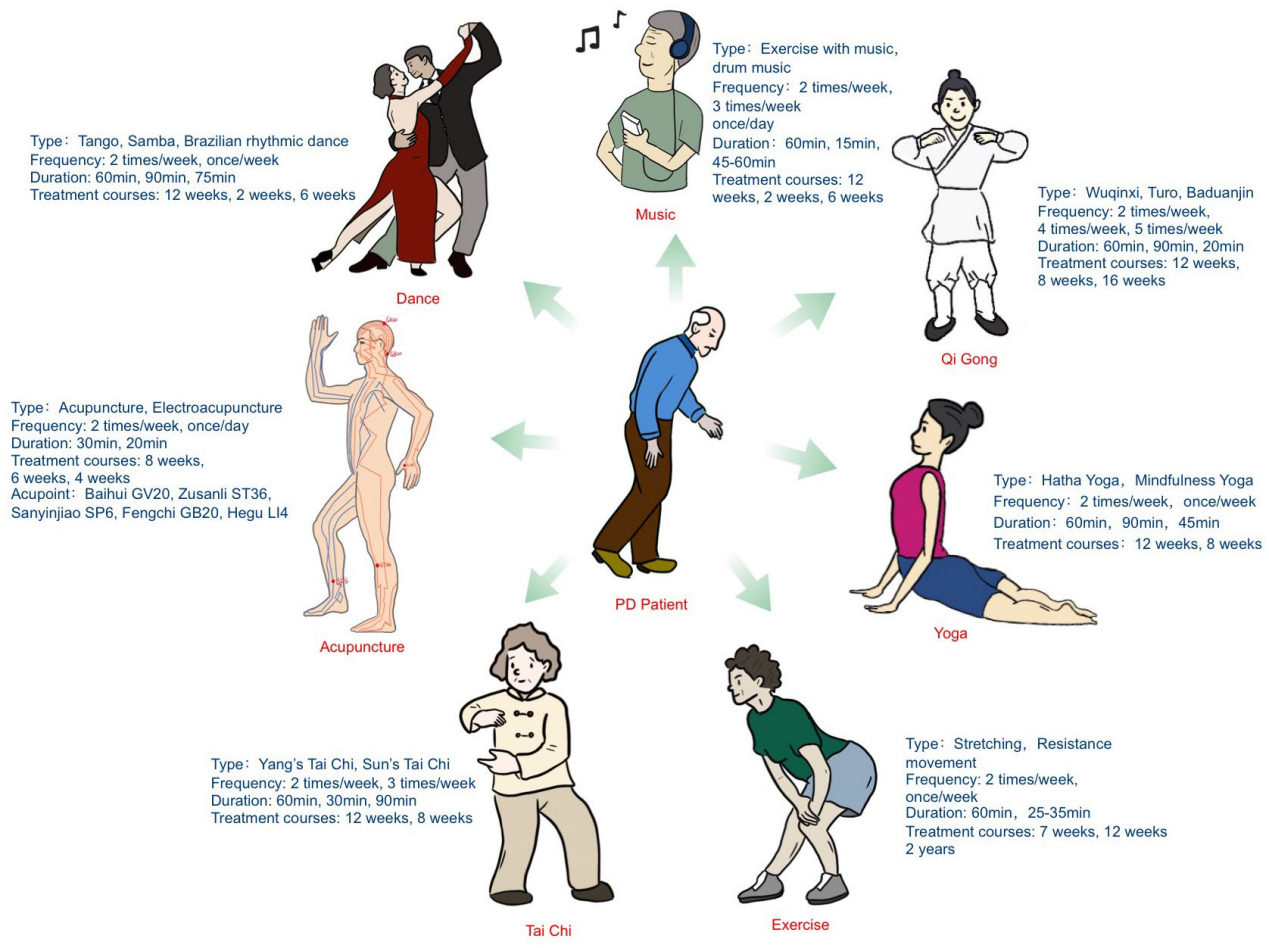
Characterization of included studies.

After selection, 58 studies (Chaiwanichsiri et al., 2011; Duncan and Earhart, 2012, 2014; Li et al., 2012, 2018, 2021, 2022; Amano et al., 2013; Cheon et al., 2013; Corcos et al., 2013; Hye-Jung et al., 2013; Nocera et al., 2013; Volpe et al., 2013; Gao et al., 2014; Chen et al., 2015; Hashimoto et al., 2015; Rios Romenets et al., 2015; Sharma et al., 2015; Zhang et al., 2015; Choi, 2016; Kluger et al., 2016; Pantelyat et al., 2016; Ventura et al., 2016; Wong-Yu and Mak, 2016; Xiao and Zhuang, 2016; Kunkel et al., 2017; Lou et al., 2017; Cheung et al., 2018; Kong et al., 2018; Kurt et al., 2018; Lee et al., 2018; Michels et al., 2018; Van Puymbroeck et al., 2018; Vergara-Diaz et al., 2018; Kalyani et al., 2019, 2020; Kwok et al., 2019, 2022; Poier et al., 2019; Rawson et al., 2019; Solla et al., 2019; Wroblewska et al., 2019; Burt et al., 2020; Cancela et al., 2020; Dos Santos Delabary et al., 2020; Elangovan et al., 2020; Jang et al., 2020; Khuzema et al., 2020; Moon et al., 2020; Pohl et al., 2020; Cherup et al., 2021; Fodor et al., 2021; Frisaldi et al., 2021; Shen et al., 2021; Brandin-de la Cruz et al., 2022; Fan et al., 2022; Nazarova et al., 2022) with 2,227 participants were included. Figure 1 illustrates the screening process, which was performed according to the PRISMA flow diagram. The basic characteristics of the literature are shown in Supplementary Table 1. Figure 2 and Supplementary Figure 2 show the risk-of-bias graph and the risk-of-bias summary, respectively, and information of the interventions is shown in Supplementary Table 2. The main treatment types, frequency, duration, and treatment courses of the included trials were as follows: qigong (types: Wuqinxi, Turo, and Baduanjin; frequency: 2 times/week, 4 times/week, and 5 times/week; duration: 60, 90, and 20 min; treatment courses: 12, 8, and 16 weeks), Tai Chi (types: Yang’s Tai Chi and Sun’s Tai Chi; frequency: 2 times/week and 3 times/week; duration: 60, 30, and 90 min; treatment courses: 12 weeks and 8 weeks), yoga (types: Hatha yoga and mindfulness yoga; frequency: 2 times/week and once/week; duration: 12 and 8 weeks; treatment courses: 60, 90, and 45 min), dance (types: Tango, Samba and rhythmic dance; frequency: 2 times/week and once/week; duration: 60, 90, and 75 min; treatment courses: 12, 2, and 6 weeks), acupuncture (types: acupuncture and electroacupuncture; frequency: 2 times/week and once/day; duration: 30 and 20 min; treatment courses: 8, 6, and 4 weeks), acupoints (e.g., Baihui GV20, Zusanli ST36, Sanyinjiao SP6, Fengchi GB20, and Hegu LI4), exercise (types: stretching and resistance movement; frequency: 2 times/week and once/week; duration: 60 and 25–35 min; treatment courses: 7, 12, and 2 years), and music (types: exercise with music and drum music; frequency: 2 times/week, 3 times/week, and once/day; duration: 60, 15, and 45–60 min; treatment courses: 12, 2, and 6 weeks). These results are shown in Figure 3.

### 3.2. Risk-of-bias assessment

Risk-of-bias plots of the literature were exported in Rev. Man 5.3, and the results were analyzed descriptively (Figure 2).

### 3.3. Effectiveness outcomes

#### 3.3.1. Evaluation of statistical inconsistency

**Figure 4:**
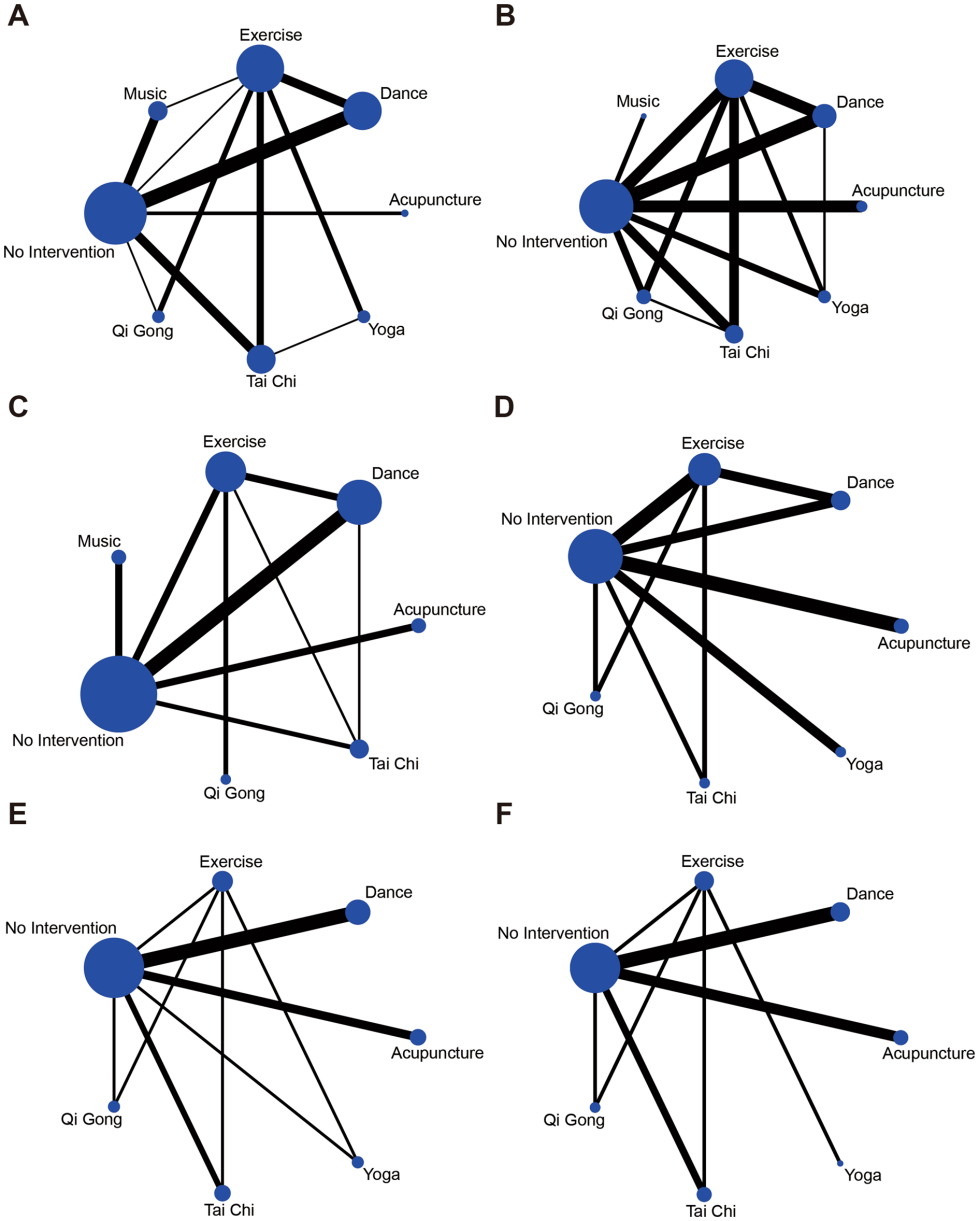
Network of analyzed comparisons. **(A)** TUG. **(B)** UPDRS-III. **(C)** PDQ-39. **(D)** UPDRS overall score. **(E)** UPDRS-I. **(F)** UPDRS-II.

A network diagram of the primary and secondary outcome eligibility comparisons under the Bayesian framework is shown in Figure 4. In the figure, the TUG test and UPDRS-III showed a reticular pattern by eight interventions, while the PDQ-39, UPDRS overall score, UPDRS-I, and UPDRS-II were surrounded by seven interventions in a reticular pattern. The thickness of the line indicates the number of studies between interventions, and the dot size indicates the sample size for that type of intervention. The closed loops prove that a consistency test was performed. Results (Supplementary Figure 3) showed no inconsistency in all measures except UPDRS-I.

### 3.4. The primary outcomes

#### 3.4.1. TUG test

TUG scores were recorded in 33 studies with 1,362 patients. The TUG test measures the patient’s ability to change position, walk, and turn and uses an objective time count as a measure. The consistency model showed that qigong performed best in the TUG test, outperforming dance (MD −3.91, 95% CI −6.90 to −0.95), Tai Chi (MD -3.54, 95% CI −6.53 to −0.69), acupuncture (MD −6.75, 95% CI -10.86 to −2.70), music (MD -3.91, 95% CI −7.49 to −0.48), exercise (MD −3.91, 95% CI −6.49 to −1.33), and no intervention (MD −5.54, 95% CI -8.28 to −2.77). Data were significant in Tai Chi (MD −1.61, 95% CI -3.00 to −0.24), dance (MD −2.00, 95% CI −3.49 to −0.44), and exercise (MD -1.63, 95% CI −3.01 to −0.23) compared with no intervention (Table 1A). In the probability ranking of the TUG test, the interventions from superior to inferior were as follows: qigong (93%), yoga (42%), Tai Chi (30%), exercise (30%), dance (25%), music (23%), no intervention (70%), and acupuncture (70%; Supplementary Figure 4).

**Table 1:** Primary outcomes.

**(A) Timed up and go test**							
Qi Gong							
−3.18(−7.23,0.82)^*^	Yoga						
−3.91(−6.90,-0.95)^*^	−0.75(−4.12,2.78)	Dance					
−3.54(−6.53,-0.69)^*^	−0.41(−3.76,3.11)	0.37(−1.59,2.28)	Tai Chi				
−6.75(−10.86,-2.70)^*^	−3.62(−7.97,0.95)	−2.84(−6.12,0.44)	−3.22(−6.48,0.12)	Acupuncture			
−3.91(−7.49,-0.48)^*^	−0.75(−4.63,3.15)	0.04(−2.76,2.64)	−0.36(−3.14,2.30)	2.86(−0.96,6.61)	Music		
−3.91(−6.49,-1.33)^*^	−0.77(−3.72,2.40)	0.03(−1.62,1.60)	−0.35(−1.97,1.30)	2.86(−0.42,6.14)	−0.00(−2.44,2.56)	Exercise	
−5.54(−8.28,-2.77)^*^	−2.40(−5.57,1.02)	−1.61(−3.00,-0.24)^*^	−2.00(−3.49,-0.44)^*^	1.24(−1.76,4.17)	−1.63(−3.98,0.84)	−1.63(−3.01,-0.23)^*^	No intervention
**(B) Unified Parkinson’s disease rating scale-III**							
Qi Gong							
4.15(−2.15,10.18)	Yoga						
4.79(−0.59,10.25)	0.65(−5.11,6.83)	Dance					
0.91(−4.07,5.74)	−3.24(−9.14,2.60)	−3.89(−9.25,1.01)	Tai Chi				
0.32(−6.17,6.54)	−3.83(−10.78,2.90)	−4.47(−10.81,1.33)	−0.65(−6.79,5.45)	Acupuncture			
−3.58(−11.55,3.92)	−7.68(−16.06,0.21)	−8.37(−16.07,-1.09)^*^	−4.47(−12.24,2.90)	−3.89(−11.65,4.03)	Music		
−0.66(−5.12,3.55)	−4.78(−9.95,0.11)	−5.45(−9.94,-1.42)^*^	−1.59(−5.32,2.08)	−0.98(−6.67,4.87)	2.88(−4.09,10.12)	Exercise	
−3.08(−7.50,1.05)	−7.20(−12.28,-2.30)^*^	−7.89(−11.92,-4.13)^*^	−4.00(−7.95,-0.06)^*^	−3.40(−8.02,1.22)	0.51(−5.82,6.92)	−2.41(−5.64,0.85)	No intervention
**(C) Parkinson’s Disease Questionnaire**							
Qi Gong					Treatments		
4.04(−9.03,17.73)	Dance				Efficacy (MD [95% Crl]), *p* < 0.05		
2.67(−11.94,17.69)	−1.41(−9.55,6.99)	Tai Chi			Efficacy (MD [95% Crl]), *p* > 0.05		
2.67(−13.36,18.01)	−1.35(−11.74,7.91)	−0.06(−12.56,11.15)	Acupuncture				
4.30(−11.98,20.20)	0.22(−10.28,10.07)	1.62(−10.80,13.09)	1.48(−10.05,13.63)	Music			
−0.50(−12.64,11.95)	−4.65(−10.23,0.89)	−3.23(−11.82,5.03)	−3.28(−12.74,7.41)	−4.81(−14.93,5.77)	Exercise		
0.91(−12.38,14.50)	−3.21(−8.24,2.16)	−1.83(−10.03,6.41)	−1.81(−9.43,7.05)	−3.33(−11.59,5.56)	1.43(−3.99,7.31)	No Intervention	

*Indicates a statistically significant value.

#### 3.4.2. UPDRS-III

We found 36 studies with 1,527 individuals reporting UPDRS-III consisting of 18 items that assess the severity of motor symptoms (trembling, stiffness, bradykinesia, gait, and postural instability) associated with PD patients. The ADDIS software analyzed the following data in the concordance model: Yoga was superior to no intervention (MD -7.20, 95% CI −12.28 to −2.30); dance was superior to music (MD −8.37, 95% CI −16.07, −1.09), exercise (MD −5.45, 95% CI −9.94 to −1.42), and no intervention (MD −7.89, 95% CI −11.92 to −4.13); and Tai Chi was superior to no intervention (MD −4.00, 95% CI −7.95 to −0.06; Table 1B). In the probability ranking of the UPDRS-III test for PD patients, the efficacy was in the order of dance (55%), yoga (39%), Tai Chi (28%), qigong (23%), acupuncture (22%), exercise (32%), no intervention (52%), and music (22%; Supplementary Figure 4).

#### 3.4.3. PDQ-39

PDQ-39 was documented in 24 studies with 883 participants. This scale can evaluate the quality of life for PD patients. In the consistency model of this measure, there was no significance in the data compared between the intervention groups (95% CI included 0;Table 1C).

### 3.5. The secondary outcomes

#### 3.5.1. UPDRS overall score

In total, 11 studies (351 participants) provided the UPDRS overall score, which is currently the most widely used comprehensive scale, including non-motor PD symptoms and the impact of motor symptoms on patients’ daily life. Data analysis showed that dance was superior to no intervention (MD −13.54, 95% CI −22.82 to −4.26); Tai Chi was superior to exercise (MD −18.22, 95% CI −30.08 to −5.93) and no intervention (MD −20.84, 95% CI −32.66 to −7.84); and acupuncture was superior to no intervention (MD −7.73, 95% CI -14.23 to −1.20; Table 2A). In the probability ranking of the UPDRS test for PD patients, the interventions from superior to inferior were Tai Chi (82%), dance (58%), qigong (29%), acupuncture (32%), yoga (32%), exercise (40%), and no intervention (70%; Supplementary Figure 5).

**Table 2:** Secondary outcomes.

**(A) Unified Parkinson’s disease rating scale overall score**						
Qi Gong					Treatments	
−1.94 (−13.51, 9.38)	Yoga				Efficacy (MD [95% Crl]), *p* < 0.05	
5.76 (−6.34, 18.12)	7.98 (−3.53, 19.35)	Dance			Efficacy (MD [95% Crl]), *p* > 0.05	
12.99 (−1.96, 27.36)	15.18 (0.63, 28.99)	7.04 (−8.47, 21.94)	Tai Chi			
0.27 (−11.40, 10.91)	2.19 (−7.22, 11.44)	−6.22 (−17.27, 5.40)	−12.77 (−26.39, 1.09)	Acupuncture		
−5.07 (−15.56, 4.92)	−3.20 (−13.76, 7.47)	−10.76 (−22.04, 0.11)	−18.22 (−30.08, −5.93)^*^	−5.37 (−15.41, 4.76)	Exercise	
−7.44 (−17.97, 1.03)	−5.57 (−12.73, 1.18)	−13.54 (−22.82, −4.26)^*^	−20.84 (−32.66, −7.84)^*^	−7.73 (−14.23, −1.20)^*^	−2.33 (−10.42,5.29)	No intervention
**(B) Unified Parkinson’s disease rating scale-I**						
Qi Gong						
0.49 (−11.69, 10.50)	Yoga					
1.91 (−11.94, 12.78)	1.53 (−6.03, 6.61)	Dance				
0.31 (−13.15, 11.33)	−0.00 (−8.10, 6.21)	−1.56 (−7.69, 5.08)	Tai Chi			
1.49 (−12.56, 12.26)	1.13 (−6.82, 6.24)	−0.48 (−5.10, 4.36)	1.10 (−5.72, 7.17)	Acupuncture		
−1.13 (−11.19, 7.40)	−1.72 (−7.14, 4.90)	−3.19 (−9.91, 5.93)	−0.85 (−5.90, 5.23)	−2.72 (−9.36, 6.53)	Exercise	
−15.50 (−19.93, −7.63)^*^	−0.37 (−6.84, 3.92)	−1.98 (−5.05, 1.31)	−0.42 (−5.86, 4.90)	−1.50 (−4.77, 2.09)	−0.77 (−6.64, 4.44)	No intervention
**(C) Unified Parkinson’s disease rating scale-II**						
Qi Gong						
1.71 (−5.74, 8.80)	Yoga					
1.75 (−3.76, 6.63)	0.15 (−7.61, 7.59)	Dance				
2.95 (−2.55, 8.04)	1.42 (−5.72, 8.20)	1.18 (−3.39, 6.14)	Tai Chi			
0.40 (−4.52, 5.55)	−1.21 (−8.65, 6.50)	−1.43 (−5.21, 3.22)	−2.62 (−7.01, 2.51)	Acupuncture		
−0.69 (−6.13, 4.48)	−2.37 (−7.58, 2.63)	−2.48 (−8.18, 3.34)	−3.77 (−8.46, 1.02)	−1.20 (−7.02, 4.14)	Exercise	
−0.75 (−5.20, 2.91)	−2.35 (−9.50, 4.41)	−2.51 (−5.54, 0.62)	−3.67 (−7.50, −0.12)^*^	−1.10 (−4.59, 1.40)	0.00 (−4.74, 4.71)	No intervention

*Indicates a statistically significant value.

#### 3.5.2. UPDRS-I

In total, 14 studies with 441 individuals reported on the UPDRS-I focusing on patients’ non-motor symptoms (including mental, behavioral, and emotional effects of patients in daily life). This index node analysis indicated *p* < 0.05, and therefore, a non-consistency model was employed. Data analysis showed that the qigong group was superior to the non-intervention group (MD −15.50, 95% CI -19.93 to −7.63; Table 2E). In the probability ranking of the PDQ-39 for PD patients, the efficacy was in the order of music (32%), dance (19%), acupuncture (18%), Tai Chi (18%), qigong (38%), no intervention (25%), and exercise (36%) (Supplementary Figure 4).

#### 3.5.3. UPDRS-II

The UPDRS-II scale is used to evaluate PD patients’ ability to live independently. The evidence from data analysis of UPDRS-II (12 studies; 356 participants) suggested that the Tai Chi group was superior to the no-intervention group (MD −3.67, 95% CI −7.50 to −0.12; Table 2C), and there was no significant difference in other intervention groups. The order of efficacy of each intervention was Tai Chi (47%), yoga (26%), dance (28%), acupuncture (23%), qigong (21%), no intervention (32%), and exercise (37%; Supplementary Figure 5).

## 4. Discussion

In this study, we presented a network meta-analysis of non-pharmacological interventions for PD, which finally included 58 studies with a total of 2,227 patients from 18 countries. It included a moderate amount of high-quality literature, some of which were of high quality. Multiple interventions were compared to each other and ranked under the primary outcome measures (the TUG test, UPDRS-III, and PDQ-39) and the secondary outcome measures (UPDRS overall score, UPDRS-I, and UPDRS-II) using Bayesian statistical methods. We found that mindfulness-based therapy was effective, that is, in addition to dance, qigong, yoga, and Tai Chi significantly improved PD symptoms as well. Therefore, mindfulness-based therapy is recommended as a priority for self-rehabilitation in PD patients in clinical practice while maintaining basic drug therapy.

This study found that qigong, yoga, Tai Chi, and dance were more effective and could improve motor function, psycho-emotional state, or daily living skills in PD patients. The first three are physical activities guided by mindfulness meditation, emphasizing the establishment of benign connections between the mind, brain, and limbs, as distinct from simple music (regulating emotions) and exercise therapy (improving motor function). The dominant interventions derived from this study (e.g., qigong, Tai Chi, and yoga) were mostly 60 min, and since these therapies require a high degree of coordination between breathing and movement, the ineffective benefits of high-intensity treatment modalities in PD patients are avoided (Schenkman et al., 2018). Studies have shown that neurotrophic factors such as BDNF, IGF-1, and VEGF can support hippocampal neuroplasticity and angiogenesis, thereby improving mood disorders and memory, but the effects vary according to different treatment frequencies (Chow et al., 2021). As a treatment modality with relatively superior efficacy data, Qigong was administered at a slightly higher frequency (2–5 times/week compared to 1–3 times/week for the other therapies), so we believe that a higher treatment frequency positively impacts efficacy.

As a part of Chinese martial arts, qigong and Tai Chi emphasize feeling the movement of “Qi” in the body (with the intention to collect Qi) and pay attention to the combination of “Qi” and movement posture. Yoga focuses on slow breathing combined with posture to achieve inner peace. While dance is a combined therapy of music, movement, and situational imagery, it is combined with the training of physical activity and cognitive skills to the accompaniment of music. Similar to other studies, this study showed that dance was most prominent for motor symptom improvement (Hao et al., 2022). However, it was not meaningfully compared with qigong, yoga, and Tai Chi. It has been shown that these three combined with mindfulness meditation can improve PD symptoms. PD is primarily caused by abnormal accumulations of Lewy bodies, reduced dopamine in the striatum, and progressive death of dopaminergic neurons in the substantia nigra pars compacta, while cortical excitability plays a key role in driving the release of the nigrostriatal dopamine (Kosillo et al., 2016). Investigation of magnetic resonance imaging data has suggested that mindfulness meditation training may be associated with cortical thickness, possibly leading to enhanced white matter integrity in the anterior cingulate cortex (ACC), greater responses in the dorsolateral prefrontal cortex (PFC), and increased activation in the parietal attention areas. This supports the effect of mindfulness meditation practice on brain attention, as it certifies the possibility of neuroplasticity in brain regions of attentional modulation (Tang et al., 2015). Qigong has also been shown to improve dopamine neurotransmission and increase regional cerebral blood flow by regulating neuroplasticity in the cortex. In PD patients, these changes may enhance basal ganglia and cortico-thalamic neural circuits, which may improve motor and non-motor symptoms (Wang et al., 2021). Considering these findings, it can be concluded that mindfulness meditation plays an important role in PD rehabilitation. In fact, acupuncture emphasizes the regulation of the mind in particular. However, modern acupuncturists lack attention to regulating patients’ emotions, which may be the reason for the poor efficacy of acupuncture. The key to the effect of acupuncture lies in the ideation compatibility between doctors and patients. Accordingly, we can probably envision better efficacy with the combination of acupuncture and mindfulness meditation.

## 5. Conclusion

This study concluded that the efficacy of non-pharmacological alternative motor−/sensory-based interventions for PD is definite and that mindfulness-based therapies such as qigong, yoga, and Tai Chi are more effective than acupuncture, exercise, and music in motor and non-motor symptoms. This provides doctors with a solid reference for developing rehabilitation programs for PD patients, and it is necessary in future to further explore the efficacy differences of different training methods within the same non-pharmacological alternative motor−/sensory-based interventions. Additionally, more high-quality studies are expected to demonstrate the relationship between mindfulness meditation and the brain, which can support the conclusions of this meta-analysis.

### 5.1. Limitations

However, our study has the following limitations. (1) Single intervention contained multiple therapies (although they belong to the same category), such as Qigong including Turo, Baduanjin, and Wuqinxi. Therefore, subgroup analysis should be performed to explore the internal differences between various qigong therapies or yoga therapies. (2) Mechanisms of mindfulness meditation and the brain have not been fully elucidated, which may affect the credibility of the conclusions of this study. (3) The practice of mindfulness meditation has certain requirements for trainers and is difficult to train, which may mislead some enthusiasts.

## Data availability statement

The original contributions presented in the study are included in the article/Supplementary material, further inquiries can be directed to the corresponding authors.

## Author contributions

SH and JW had full access to all the data in the study and took responsibility for the integrity of the data and the accuracy of the data analysis. SH, WF, and JW contributed to the concept and design. SH and WF were involved in the acquisition, analysis, or interpretation of data. SH, WF, JW, HL, JZ, TW, YH, GL, and ML contributed to the drafting of the manuscript. ML, GL, and YH critically revised the manuscript for important intellectual content. SH, WF, and JW performed statistical analysis. ML provided administrative, technical, or material support. ML, GL, and YH supervised the study. All authors contributed to the article and approved the submitted version.

## Funding

This research was partially supported by the National Natural Science Foundation of China (no. 82004450). The funder had no role in the design and conduct of the study; collection, management, analysis, and interpretation of the data; preparation, review, or approval of the manuscript; and decision to submit the manuscript for publication.

## Conflict of interest

The authors declare that the research was conducted in the absence of any commercial or financial relationships that could be construed as a potential conflict of interest.

## Publisher’s note

All claims expressed in this article are solely those of the authors and do not necessarily represent those of their affiliated organizations, or those of the publisher, the editors and the reviewers. Any product that may be evaluated in this article, or claim that may be made by its manufacturer, is not guaranteed or endorsed by the publisher.
