# Supplementary material for: Whether mindfulness-guided therapy can be a new direction for the rehabilitation of patients with Parkinson’s disease: a network meta-analysis of non-pharmacological alternative motor-/sensory-based interventions
Source: Front Psychol. 2023 Sep 14;14:1162574. doi: 10.3389/fpsyg.2023.1162574 (PMC10540073; doi:10.3389/fpsyg.2023.1162574)
Supplement: Supplementary file 1 [file Data_Sheet_2.PDF]

Supplementary Material

Whether mindfulness-guided therapy can be a new direction for the rehabilitation of patients with Parkinson 's disease: A network meta-analysis of non-pharmacological alternative motor-based interventions

Shenglan He [1#], Wanyi Fang [1#], Jiaoyang Wu [1#], Hang Lv[2], Yuling Shi[3], Jueyu Zhang [1,4], Tunyi Wang [1], Yingjie Huang [1 *], Guangyao Li [1 *], Min Li [1 *]

[1]Medical College of acupuncture Moxibustion and Rehabilitation, Guangzhou University of Chinese Medicine, Guangzhou, China.

[2] The Affiliated Traditional Chinese Medicine Hospital of Guangzhou Medical University

[3] Guangdong Second Traditional Chinese Medicine Hospital (Fifth Clinical Medical College of Guangzhou University of Chinese Medicine).

Corresponding author:

corresponding to: Yingjie Huang ， Guangyao Li，Min Li

Address: [No. 12 Airport Road, Baiyun District, Guangzhou City, Guangdong Province, People's Republic of China.]

Telephone: [020-39358430]

Email: [Yingjie Huang: 2579726475@qq.com; Guangyao Li: doctorli666888@163.com; Min Li: professorlimin@163.com]

1 Supplementary Data

No Supplementary Data

2 Supplementary Figures and Tables

2.1 Supplementary Figures

Supplementary Figure 1. Search Strategy

#1 Parkinson [MeSH Terms]
#2 Acupuncture [MeSH Terms]
#3 ((((((((((((Acupuncture Therapy [Title/Abstract]
OR (electro － acupuncture［Title /Abstract］)
OR (Zhenjiu［Title /Abstract］)
OR (Zhenci［Title / Abstract］)
OR (Dianzhen［Title /Abstract］)
OR (scalp － acupuncture［Title /Abstract］)
OR (auricular acupuncture ［Title /Abstract］)
OR (acupuncture technique［Title / Abstract］)
OR (warm acupuncture［Title /Abstract］)
OR (abdominal acupuncture［Title /Abstract］)
OR (fire needle therapy ［Title /Abstract］)
OR (bee-sting therapy ［Title /Abstract］)
OR (moxibustion ［Title /Abstract］)
#4 #2 OR #3
#5 Qi Gong [MeSH Terms]
#6 (((((QiGong［Title /Abstract］
OR (Qi-Gong［Title /Abstract])
OR (qigong［Title /Abstract］)
OR (qi-gong［Title /Abstract］)
OR (qi gong［Title /Abstract］)
#7 #5 OR #6
#8 Yoga [MeSH Terms]
#9 Dance [MeSH Terms]
#10 (Dancing［Title /Abstract])
#11 #9 OR #10
#12 Tai Chi [MeSH Terms]
#13((((((((Tai-ji［Title /Abstract]
OR Chi, Tai［Title /Abstract])
OR Tai Ji Quan［Title /Abstract])
OR Ji Quan, Tai［Title /Abstract])
OR Quan, Tai Ji［Title /Abstract])
OR Taiji［Title /Abstract])
OR Taijiquan［Title /Abstract])
OR T'ai Chi［Title /Abstract])
OR Tai Chi Chuan［Title /Abstract])
#14 #12 OR #13
#15 music [MeSH Terms]
#16 ((((((((Rap Music［Title /Abstract]
OR Hip Hop Music［Title /Abstract])
OR Hop Music, Hip［Title /Abstract])
OR Jazz Music［Title /Abstract])
OR Classical Music［Title /Abstract])
OR Song［Title /Abstract])
OR Vocal Melody［Title /Abstract])
OR Melodies, Vocal［Title /Abstract])
#17 #15 OR #16
#18 Exercises [MeSH Terms]
#19 (((((((((Physical Activity［Title /Abstract]
OR Activities, Physical［Title /Abstract])
OR Exercise, Physical［Title /Abstract])
OR Acute Exercise［Title /Abstract])
OR Exercise, Acute［Title /Abstract])
OR Exercise, Isometric［Title /Abstract])
OR Isometric Exercise［Title /Abstract])
OR Exercise, Aerobic［Title /Abstract])
OR Exercise Training［Title /Abstract])
#20 #18 OR #19
#21 Meditation [MeSH Terms]
#22 (Mindfullness［Title /Abstract])
#23 #21 OR #22
#21 #1 AND #4 OR #7 OR #8 OR #11 OR #14 OR #17 OR #20 OR #23

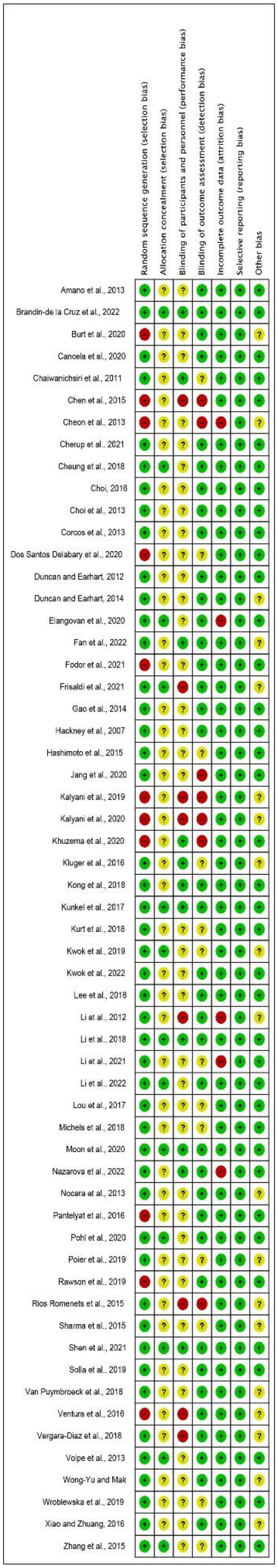

Supplementary Figure 2. Risk of Bias Summary

Name	Direct Effect	Indirect Effect	Overall	P-Value
A, G	4.26 (1.22, 7.41)	3.01 (-2.09, 8.17)	3.91 (1.33, 6.49)	0.68
A, H	4.70 (-0.31, 9.65)	5.97 (2.58, 9.43)	5.54 (2.77, 8.28)	0.67
B, D	-2.30 (-9.27, 4.91)	0.74 (-2.80, 4.26)	0.41 (-3.11, 3.76)	0.39
C, G	1.20 (-0.79, 3.11)	-1.69 (-3.71, 0.51)	-0.03 (-1.60, 1.62)	0.06
C, H	0.83 (-0.56, 2.28)	3.69 (1.13, 6.10)	1.61 (0.24, 3.00)	0.06
D, G	-0.46 (-2.74, 1.84)	1.26 (-1.04, 3.63)	0.35 (-1.30, 1.97)	0.28
D, H	2.37 (0.50, 4.13)	1.15 (-1.56, 3.97)	2.00 (0.44, 3.49)	0.45
F, G	0.99 (-2.87, 4.92)	-0.85 (-4.26, 2.44)	0.00 (-2.56, 2.44)	0.46
F, H	0.88 (-2.31, 4.01)	2.78 (-1.34, 7.01)	1.63 (-0.84, 3.98)	0.45
G, H	2.73 (0.72, 4.79)	0.88 (-0.84, 2.57)	1.63 (0.23, 3.01)	0.16

TUG

Name	Direct Effect	Indirect Effect	Overall	P-Value
A, D	-1.45 (-11.97, 9.00)	-0.73 (-6.45, 5.19)	-0.91 (-5.74, 4.07)	0.90
A, G	3.13 (-2.77, 9.05)	-1.64 (-7.84, 4.46)	0.66 (-3.55, 5.12)	0.26
A, H	0.95 (-5.30, 7.15)	5.37 (-0.72, 11.51)	3.08 (-1.05, 7.50)	0.30
B, G	6.22 (-0.71, 13.30)	3.04 (-4.85, 10.84)	4.78 (-0.11, 9.95)	0.53
B, H	5.74 (-1.25, 12.55)	9.10 (1.26, 16.94)	7.20 (2.30, 12.28)	0.51
C, G	2.54 (-3.58, 8.64)	8.60 (3.03, 14.79)	5.45 (1.42, 9.94)	0.14
C, H	9.68 (5.37, 14.42)	3.76 (-3.25, 10.85)	7.89 (4.13, 11.92)	0.15
D, G	2.18 (-2.74, 7.17)	1.52 (-4.33, 7.53)	1.59 (-2.08, 5.32)	0.85
D, H	3.80 (-1.53, 9.32)	4.64 (-1.04, 10.51)	4.00 (0.06, 7.95)	0.82
G, H	3.83 (-1.52, 9.11)	1.16 (-2.93, 5.24)	2.41 (-0.85, 5.64)	0.42

UPDRS-III

Name	Direct Effect	Indirect Effect	Overall	P-Value
C, D	1.55 (-13.09, 16.29)	1.01 (-9.84, 11.68)	1.20 (-7.30, 9.56)	0.95
C, G	8.27 (1.94, 13.97)	-1.21 (-7.84, 7.01)	4.60 (-0.91, 10.61)	0.07
C, H	-0.22 (-5.40, 5.60)	9.41 (1.16, 16.11)	3.12 (-2.28, 8.45)	0.07
D, G	3.00 (-11.52, 17.30)	3.62 (-7.31, 15.17)	3.41 (-4.94, 12.13)	0.93
D, H	2.64 (-9.73, 15.37)	1.59 (-9.97, 12.73)	1.80 (-6.41, 10.28)	0.88
G, H	2.46 (-4.22, 7.77)	-6.97 (-13.49, 0.87)	-1.55 (-7.32, 4.01)	0.08

PDQ-39

Name	Direct Effect	Indirect Effect	Overall	P-Value
A, G	12.91 (-9.53, 30.10)	0.45 (-13.17, 14.10)	5.07 (-4.92, 15.56)	0.30
A, H	4.36 (-5.07, 13.98)	16.49 (-2.00, 36.21)	7.44 (-1.03, 17.97)	0.31
C, G	9.11 (-2.68, 24.50)	10.70 (-1.91, 25.41)	10.76 (-0.11, 22.04)	0.78
G, H	4.78 (-4.05, 13.00)	-6.37 (-21.85, 9.81)	2.33 (-5.29, 10.42)	0.20

UPDRS overall score

Name	Direct Effect	Indirect Effect	Overall	P-Value
A, G	-2.37 (-10.54, 5.71)	17.10 (11.66, 22.77)	N/A	0.00
A, H	16.39 (12.03, 20.86)	-2.98 (-11.78, 5.73)	N/A	0.00
B, G	3.36 (-6.46, 13.11)	-5.13 (-18.34, 6.36)	N/A	0.22
B, H	-0.45 (-10.28, 9.29)	7.94 (-3.90, 21.20)	N/A	0.23
D, G	0.91 (-9.41, 11.31)	-4.97 (-18.71, 7.27)	N/A	0.41
G, H	0.87 (-10.14, 11.64)	3.54 (-4.70, 13.18)	N/A	0.64

UPDRS-I

Name	Direct Effect	Indirect Effect	Overall	P-Value
A, G	2.79 (-6.28, 11.44)	0.19 (-8.03, 7.14)	0.69 (-4.48, 6.13)	0.63
A, H	0.54 (-4.31, 5.44)	2.64 (-8.20, 13.42)	0.75 (-2.91, 5.20)	0.72
D, G	3.86 (-1.47, 9.27)	5.61 (-4.66, 16.58)	3.77 (-1.02, 8.46)	0.74
G, H	1.98 (-3.75, 7.76)	-2.29 (-7.63, 3.48)	-0.00 (-4.71, 4.74)	0.21

UPDRS-II

Supplementary figure 3:Node-split model:A:qigong B:youga C:dance D:taichi E:acupuncture F:music G:exercise H:no intervention

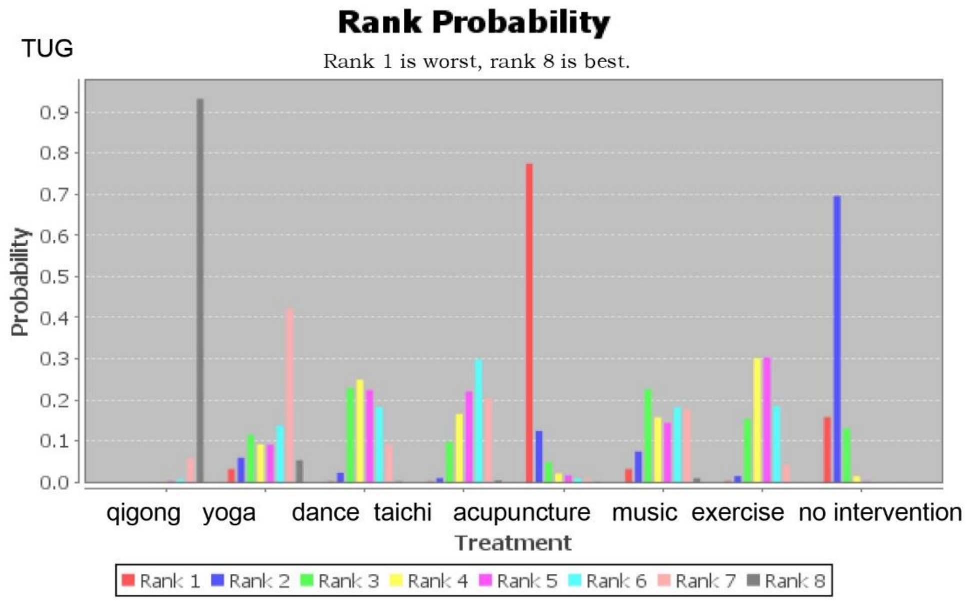

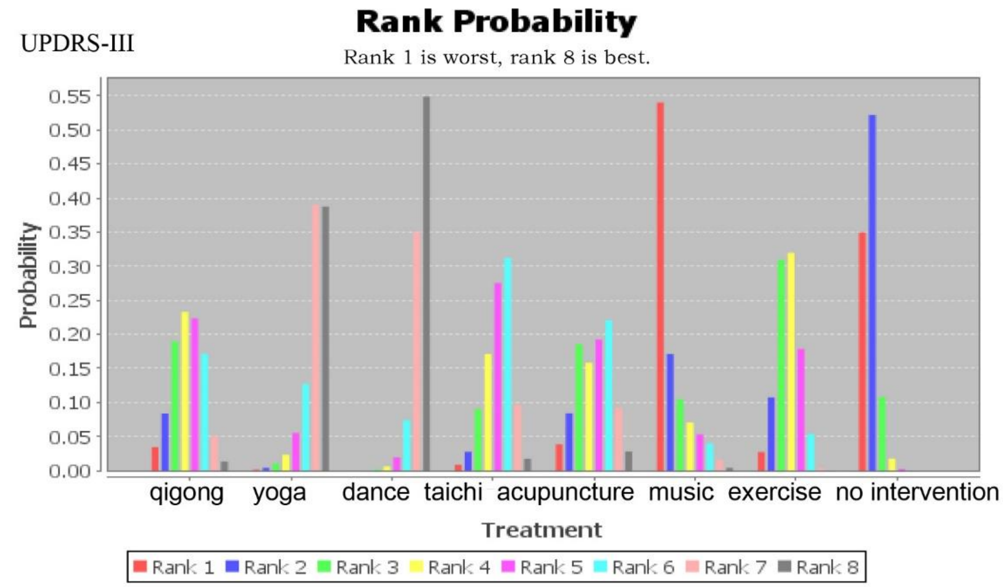

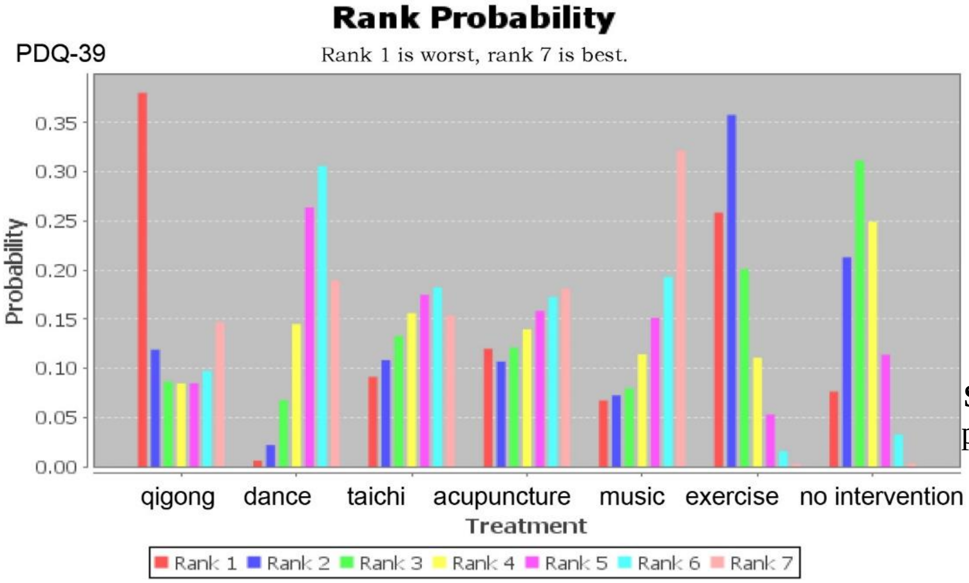

Supplementary Figure 4: Rank probability of primary outcomes

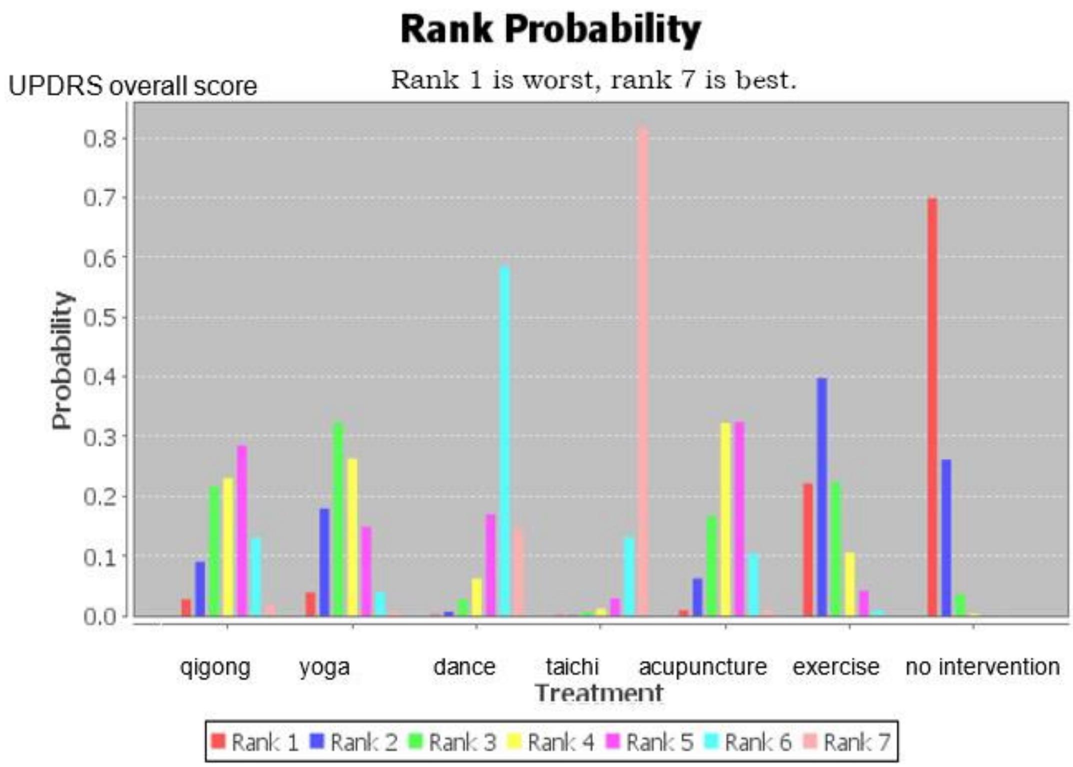

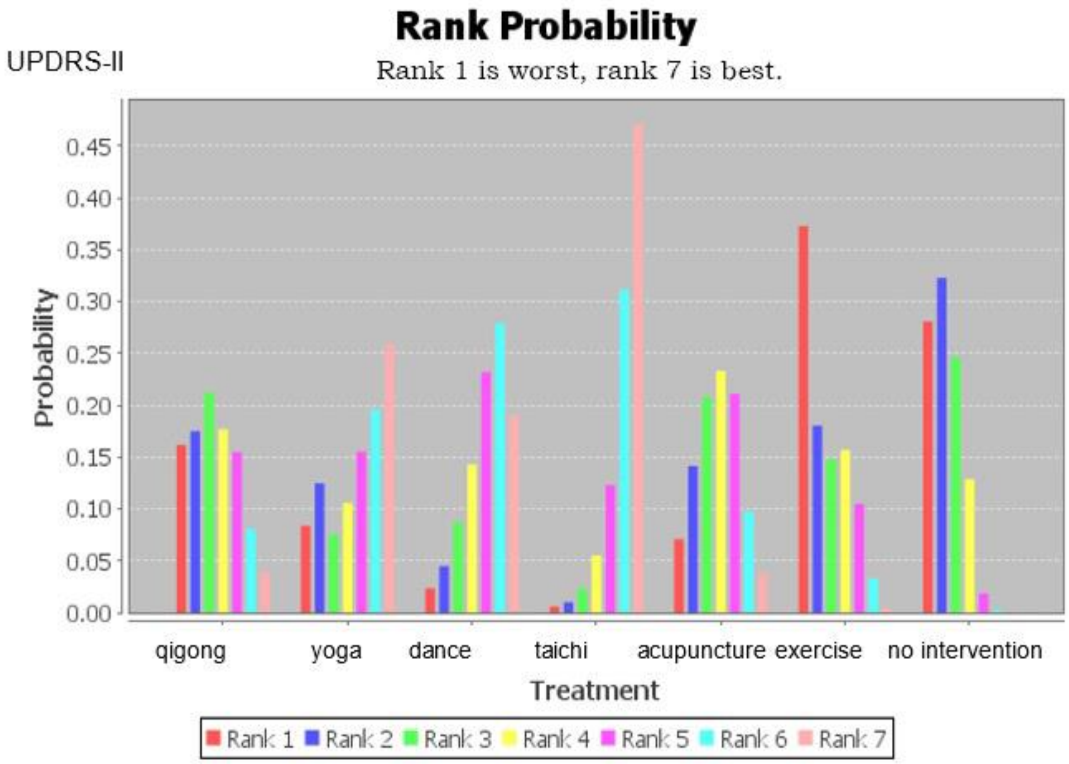

Supplementary Figure 5: Rank probability of secondary outcomes

2.2 Supplementary Tables

Supplementary Table 1: Information of Studies

Study ID	Country	Sample size(EG/CG)	Intervention		intervention parameter	Days	Outcome
Chen et al., 2015	China	40(20/20)	acupuncture	no intervention	15min. Twice per week	126	②③④⑤
Volpe et al., 2013	Italy	24(12/12)	dance	exercise	1.5h. Once per week	180	⑤⑥
Ventura et al., 2016	USA	15(7/8)	dance	no intervention	1.25h. Once per week	70	①⑥
Kunkel et al., 2017	UK	46 (31/15)	dance	no intervention	60min. Tiwce per week	70	①⑤⑥
Sharma et al., 2015	USA	13(8/5)	yoga	no intervention	Twice a week	84	②
Poier et al., 2019	Germany	29(14/15)	dance	taichi	60min. Once per week	70	⑥
Corcos et al., 2013	USA	48(24/24)	exercise	no intervention	twice per week(first 6 months)<br><br>Once per week	730	⑤⑥
Kong et al., 2018	Singapore	34(18/16)	acupuncture	no intervention	20min. Twice per week	35	⑤⑥
Li et al., 2018	CHINA	25 (11/14)	acupuncture	no intervention	30min. Twice per week	84	④⑤
Duncan and Earhart, 2014	USA	10(5/5)	dance	no intervention	60in. Twice per week	730	①③④⑤
Moon et al., 2020	USA	17(8/9)	qigong	no intervention	15-20min. Twice per day	84	③④⑤⑥

Dos Santos Delabary et al., 2020	Brazil	18(12/6)	dance	exercise	60min. Twice per week	84	①⑤
Li et al., 2022	CHINA	40(20/20)	qigong	exercise	90min. Twice per week	84	①②⑤⑥
Kalyani et al., 2020	Australia	33 (17/16)	dance	no intervention	60min. Twice per week	90	①③④⑤
Michels et al., 2018	USA	13(9/4)	dance	no intervention	60min. Once a week	70	①②④⑤⑥
Pantelyat et al., 2016	USA	18 (8/10)	music	exercise	45-60min. Twice per week	42	①⑤⑥
Xiao and Zhuang, 2016	CHINA	89 (45/44)	qigong	exercise	45-min. Four times per week<br>daily walking 30 min	180	①⑤
Khuzema et al., 2020	India	27(9/9/9)	taichi	yoga	30-40min	56	①②⑤⑥
Lou et al., 2017	CHINA	74(36/38)	taichi	no intervention	90 min. 4 times per week	49	①
Frisaldi et al., 2021	Italy	38(19/19)	dance	no intervention	1h dance class+1h conventional physiotherapy. Three times per week	35	①⑤⑥
Fan et al., 2022	CHINA	64 (32/32)	acupuncture	no intervention	30-min. Three times per week	56	②③⑥
Cancela et al., 2020	Spain	14(7/7)	exercise	exercise	25-35min. Once per week	56	②⑤⑥
Kurt et al., 2018	Turkey	40(20/20)	taichi	exercise	60min. 6 times per week	56	①⑤⑥

Hashimoto et al., 2015	Japan	46(15/14/14)	dance	exercise	60min sessions per week	84	①②
Brandin-de la Cruz et al., 2022	Spain	33 (18/15)	acupuncture	no intervention	1Hz over 1min per MTrP	once	①②
Li et al., 2021	CHINA	31 (15/16)	qigong	no intervention	60min. 5 times per week	84	①⑤
Kwok et al., 2022	China	52（26/26)	yoga	exercise	90min. Once a week	56	③④
Kwok et al., 2019	China	138(71/67)	yoga	exercise	90min. Once a week	56	①⑤
Zhang et al., 2015	CHINA	40(20/20)	taichi	exercise	60min. Twice per week	84	①⑤
Gao et al., 2014	CHINA	76(37/39)	taichi	no intervention	60min. Three times per week	84	①⑤
Hackney et al., 2007	USA	19(9/10)	dance	exercise	1h. Twice per week	91	①⑤
Choi, 2016	korea	20(11/9)	taichi	no intervention	60min. Three times per week	84	①②
Cheung et al., 2018	USA	20 (10/10)	yoga	no intervention	60min. Twice per week	84	⑤
Rawson et al., 2019	USA	59(36/23)	dance	exercise	60min. Twice per week	84	⑤⑥
Van Puymbroeck et al., 2018	USA	27 (15/12)	yoga	-	twice per week	56	⑤
Jang et al., 2020	Korea	25(12/13)	acupuncture	no intervention	twice per week	28	①④⑤
Pohl et al., 2020	Sweden	46(26/20)	music	no intervention	60min. Twice per week	84	①⑥

Elangovan et al., 2020	USA	18(9/9)	yoga	no intervention	60min. Twice per week	84	⑤
Kalyani et al., 2019	Australia	33 (17/16)	dance	no intervention	60min. Twice per week	84	③④⑤⑥
Wong-Yu and Mak.,2016	Hong Kong	80(41/39)	exercise	no intervention	2h. Once per week	56	①⑤⑥
Fodor et al., 2021	Romania	32(16/16)	music	no intervention	2.5h/day	14	⑥
Duncan and Earhart, 2012	USA	52 (26/26)	dance	no intervention	1h.Twice per week	365	③④
Kluger et al., 2016	USA	94(47/47)	acupuncture	no intervention	30min. Twice per week	42	⑤⑥
Solla et al., 2019	Italy	19 (10/9)	dance	no intervention	90min.Twice per week	84	①⑤
Li et al., 2012	USA	130 (65/65)	taichi	exercise	60min. Twice per week	168	①⑤
Nocera et al., 2013	USA	21(15/6)	taichi	no intervention	60min. 3 times per week	112	⑥
Vergara-Diaz et al., 2018	USA	27 (12/15)	taichi	no intervention	60min. Twice per week	180	①⑤⑥
Rios Romenets et al., 2015	Canada	33(18/15)	dance	no intervention	60min. Twice per week	84	①⑤⑥
Nazarova et al., 2022	CHINA	30(15/15)	acupuncture	no intervention	50/100HZ. 30min. Twice per week	56	③④⑤
Amano et al., 2013	USA	21(12/9)	taichi	qigong	60min. Twice a week.<br>60min. Three times per week	112	⑤
Burt et al., 2020	Canada	30(15/15)	music	no intervention	15 min. Three times per week	84	⑤

Cheon et al., 2013	Korea	23(9/7/7)	taichi	exercise	50-65min. Three times per week	56	③
Shen et al., 2021	Korea	30(15/15)	qigong	exercise	90min. Twice per week	84	①⑤
Wroblewska et al., 2019	Poland	40(20/20)	exercise	no intervention	60min. Twice per week	84	①③④⑤
Choi et al., 2013	Korea	20(11/9)	taichi	no intervention	60min. Three times per week	84	①
Chaiwanichsiri et al., 2011	Thailand	20(10/10)	music	no intervention	30min. Three times per week	56	①
Lee et al., 2018	Korea	41 (25/16)	dance	no intervention	60min. Twice per week	56	③④⑤
Cherup et al., 2021	USA	33 (15/18)	yoga	exercise	45min. Twice per week	84	①

① TUG②UPDRS③UPDRS I④UPDRS II⑤UPDRS III⑥PDQ-39

Supplementary Table 2: Information of interventions

Interventions	Number of studies	Average time(range)	Frequency(range)	Course(range)	Total time
acupuncture	8	26min(15-30min)	twice a week(2-3times/w)	61days(35-126d)	416min
dance	17	64min(60-90min)	1.76times/week(1-3times/w)	137days(35-730d)	2204min
qigong	6	68min(15-90min)	3 times/week(2-5times/w)	105days(84-180d)	3069min
taichi	13	56min(50-90min)	2.8 times/week(2-6times/w)	92days(49-180d)	2060min
yoga	8	63min(45-90min)	1.71times/week(1-2times/w)	70days(56-84d)	1071min
exercise	20	67min(25-120min)	twice a week(1-6times/w)	120days(42-730d)	2296min
music	5	61.5min(15-150min)	3.4 times /week(2-7times/w)	140days(14-84d)	4182min
